# Electrostatic separator of cannabis trichomes: an innovative approach to extraction

**DOI:** 10.1186/s42238-025-00281-z

**Published:** 2025-05-04

**Authors:** Charles MacGowan, Alex Martynenko

**Affiliations:** 1 Sambo Creeck Filtration, Miami, FL 33122 USA; 2 Faculty of Agriculture, Dalhousie University, Truro, NS B2 N 5E3 Canada

**Keywords:** Glandular trichomes, Dry separation, Separability, Free-fall, Electrostatic, Cannabis

## Abstract

**Background:**

Efficient separation of trichomes from plant material is critical for producing high-quality cannabis extracts. Traditional methods of separation, such as wet and dry fractionation, use the difference in mechanical properties (size, density, specific gravity) between cannabis trichomes and plant biomass. However, these methods were developed to process small quantities of raw material and are very much labor-intensive. On the other hand, the quickly growing cannabis industry requires fully automated, scalable technology for the efficient extraction of valuable trichomes from the large volume of plant biomass. Therefore, our research aimed to develop a scalable method and equipment for trichome separation.

**Methods:**

We have measured electrical properties of trichomes and plant biomass, namely electrical conductivity, dielectric permeability and particle charge, using a Keithley 6517B electrometer in a Faraday cage in controlled conditions. The fundamental forces acting on the charged particle in a strong electric field were analyzed using the theory of electrostatics.

**Discussion:**

It was found that plant biomass had a positive electric charge, while trichomes had a negative electric charge. A difference in electric charge between trichomes and plant biomass suggested an electrostatic method of separation. This paper explores the application of electrostatic separation as a novel, sustainable, and efficient method for isolating cannabis trichomes. A new concept for a free-fall electrostatic separator for cannabis trichomes is proposed, and the prototype of the electrostatic separator is described. This method minimizes the need for manual labor, allowing the separation of cannabis trichomes to a desirable purity. The separator is scalable from 1 to 100 kg/hour and can be fully automated.

## Introduction

Cannabis trichomes are glandular structures that play a crucial role in the quality and potency of cannabis products. The function of glandular trichomes is to secrete complex metabolites and phytochemicals, such as terpenoids, phenylpropanoids, flavonoids, etc., which are accumulated in trichome heads. As a result, trichomes contain large amounts of secondary metabolites, which are valuable for the pharmaceutical and nutraceutical industries. The separation of trichomes from plant biomass is an extremely important industrial operation.

One method to separate glandular trichomes from plant biomass is wet fractionation through mechanical agitation of plant flowers in ice water (Delp, 2000). Ice water causes the resins to become brittle, while the remaining plant material remains more flexible, which allows separation based on the difference in specific gravity. The drawback of the ice-water method is the loss of some valuable aromatic components, such as terpenes and light oils, in the water. Another drawback of this method is the need for subsequent dewatering to avoid fungal propagation. Dewatering is an expensive process, making it challenging to deploy advanced wet fractionation techniques in commercial applications. Also, wetting may lead to the alteration/degradation of the organoleptic properties of trichomes.

An alternative approach is dry separation based on some differences in the physical or surface properties of the components. These may be physical/mechanical, magnetic or electrical. Since plant material does not possess any magnetic properties, it is not considered for trichome extraction.

Mechanical separation based on the tumbling or sieving of the plant biomass to screen out glandular trichomes (Watts & Amovick, 2019). The method requires pre-freezing of plant biomass with a liquid freezing agent, such as liquid CO_2,_ with the next separation on the centrifuge, using the difference in density between trichome heads and plant matter. The disadvantage of this method is that the process is extremely sensitive to operating parameters such as temperature, humidity, and process duration. To get the high-purity product, a cold, dry atmosphere and a short processing time are required. However, under these conditions, the yield is low. An increase in yield causes a loss of purity, which requires further product purification from contaminants. Also, mechanical separation can damage delicate trichomes, reduce cannabinoid and terpene content, or introduce contaminants.

Another method of dry separation, so-called “static tech”, is based on the mechanical rubbing of dry plant powder on the nylon sift screen (Philips 2022). It is believed that due to the friction, the plant biomass and trichomes acquire different charges, which allows the hand-pick collection of trichome heads with nitrile gloves or parchment paper. However, this method is labor-intensive and not scalable. It is recognized that this separation technique requires highly trained personnel to reach the desired quality and purity of the product. At the same time, the “static tech” technique highlighted the significance of triboelectric charging in trichomes and plant biomass, drawing attention to the electrical properties of cannabis trichomes for their separation. A difference in electric properties between trichomes and plant biomass suggested an electrostatic method of separation.

Electrostatic separation is based on the differential attraction or repulsion of charged particles under the influence of a very high electric field (Ralston 1961). Historically, electrostatic separation has roots in mineral processing, where it plays a crucial role in separating conductive and non-conductive minerals (Mazumder et al. 1994; Kelly and Spottiswood 1989a, b). Early adopters found it invaluable in industries where traditional wet separation techniques were either impractical or economically unfeasible. It was demonstrated that electrostatic separators could achieve high-purity separation, paving the way for large-scale commercial applications (Inculet 1985).

According to the engineering design, electrostatic separators could be distinguished into three categories: (1) electroconductive rotating drum separators, (2) conveyor belt separators, (3) free-fall separators. All of them contain a system to charge particles, an externally generated electric field for separation, and a system to convey particles in and out of the separation apparatus. Electrical charging can occur by one of multiple methods, including conductive or non-conductive induction, triboelectric charging, and corona charging.

A rotating drum electrostatic separator is preferable for separating particles with different electrical conductivity. Multiple variations and geometries are used for conductive drum separators, but they generally operate on similar principles (Jordan and Weaver, 1978). Feed particles are dispersed onto a rotating drum that is electrically grounded and then charged by either induction or corona discharge. The separation is based on the differences in discharge time. Electroconductive particles quickly give up their charge to the surface of the grounded drum. The rotation of the drum creates centrifugal force, which, in combination with gravitational force, facilitates the release of these particles from the drum surface to the primary bin. In contrast, non-conductive particles retain their electrical charge, which will keep them attracted to the drum surface. Eventually, they will be brushed from the surface into a secondary bin. In some applications, several intermediate bins are introduced between primary and secondary bins.

Conveyor belt separators use the same principle of electrostatic attraction. In this case, the separation of charged particles is based on electrostatic and gravitational forces, with no centrifugal force involved. Both drum and conveyor belt separators work well for particles larger than 1 mm, but not for powders, where particle–particle adhesion forces become significant. For fine particles, adhesion forces dominate over electrostatic forces. They stick to each other rather than to the electrode surface. This creates a problem of powder conglomeration, which makes separation impossible.

The conglomeration problem is addressed by free-fall electrostatic separation, where fine particles are suspended in the air in a diffused state. This arrangement minimizes particle–particle interaction. However, in this case, each particle should be individually charged. The preference is given to triboelectric charging due to its ability to suspend particles in the turbulent airflow. Matsusaka et al. (2010) reviewed different mechanisms of triboelectric charging of fine powders, including contact charging, charge relaxation, wall friction, repeated impacts of a single particle, and particle charging in gas-solids pipe flow. It is worth noting that triboelectric charging depends on the environmental conditions, such as temperature and humidity, which should be strictly controlled.

A typical free-fall electrostatic separator is described by Bendimerad et al. (2014). It contains a cyclone or rotating vibrator for mechanical disconnection and triboelectric charging of individual particles. Airflow delivers charged particles to the separation chamber, where they fall under gravity in a strong electric field. The acquired charge determines the free-falling trajectory of the particle in the unidirectional electric field.

The limited application of electrostatic separation for plant biomass could be explained by the comparatively low commercial value of most agricultural commodities. Also, plant materials are non-conductive, which makes traditional electrostatic separation difficult or even impossible. For this reason, other principles of electrostatic separation, based on the difference in triboelectric charges and dielectric properties, have been developed (Carpenter, 1951). Early applications showed the versatility of electrostatic separation of plant materials as a good alternative to wet separation (Harmond et al. 1961). For example, a rotating drum separator was successfully used for seed cleaning (Mezynski 1965). A free-fall electrostatic separation of plant materials was patented by Rajabzadeh et al. (2015).

One of the challenges of free-fall powder separation is particle-electrode collision on the planar electrodes, which decreases separation efficiency. To mitigate this problem, Eddine et al. (2025) proposed a segmented electrode design. A non-homogeneous electric field in this design facilitated the deflection of oppositely charged particles, improving the separation efficiency.

The information on electrostatic separation of plant-based powders is very limited. Wang et al. (2014) explored feasibility of free-fall separation of proteins and carbohydrates from yellow pea flour, using a lab-scale electrostatic separator. The particle trajectories are deflected in the electric field according to the polarity and the amount of charge (Wang et al. 2015). However, even with low throughput, it did not achieve the desired yield and quality of separation (Xing et al. 2020).

Electrostatic separation of trichomes is difficult due to the heterogeneous feedstock and small differences between cannabis trichomes and plant biomass. Hence, the design of an electrostatic separator for cannabis presents a challenge and significant innovation. This paper summarizes three years of research and development of a conceptually new technique of electrostatic separation of cannabis trichomes, based on the unique combination of electrical, mechanical and chemical properties. The scientific contribution of this research is related to the first-time measurements of trichomes'electric charge. The practical contribution of this research is related to using the difference in electric charge for the separation of trichomes from plant biomass. Based on the principles of electrostatic separation and system design, we have proposed a scalable technology for the efficient extraction of trichomes from the large volume of plant biomass. First in the world, we designed and tested the pilot-scale electrostatic separator of cannabis trichomes.

## Materials and methods

### Particle charge measurements

**Fig. 1:**
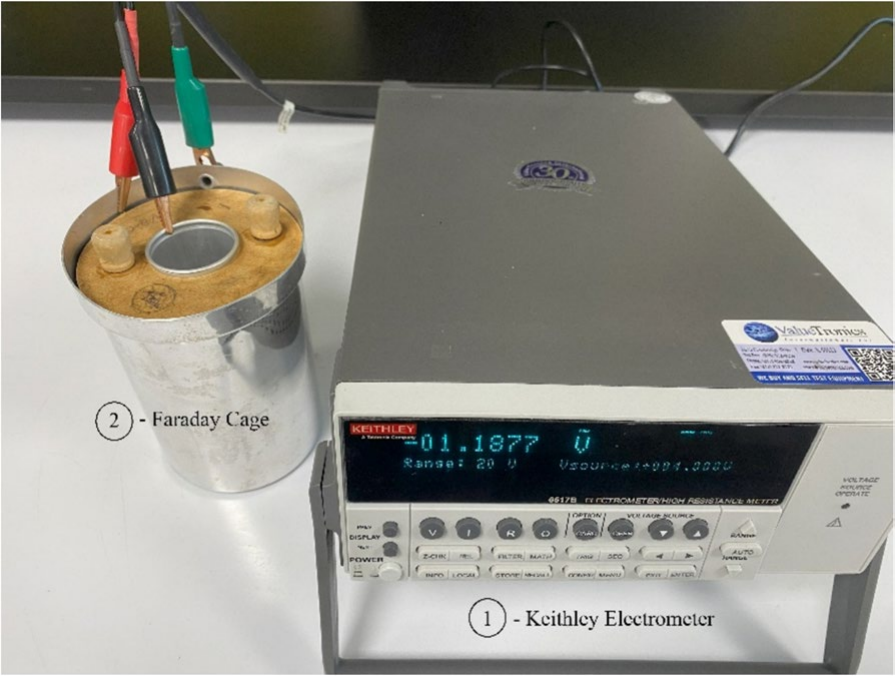
Charge measurement with Keithley 6517B electrometer (1) and Faraday cage (2)

In our experiments, we used samples of ground cannabis material with different volumetric fractions of trichomes. The electric charge of the samples was measured in a Faraday cage using a Keithley 6517B electrometer (Tektronix Inc., Beaverton, OR, USA). The measurement setup is shown in Fig. 1. All measurements were conducted at a temperature of 15 °C and a relative humidity of 30–34%.

The Faraday cage was made of two aluminum cylinders, separated by a dielectric material, creating a cylindrical capacitor with 2.4 nF capacitance. An internal cylinder of 1″ diameter was filled either with plant biomass or trichome material for charge measurements. The charge was calculated from the voltage readings, using the formula:

1 $$Q=CV$$

The nature of electric charge could be related to the chemical composition of plant biomass (Wang et al. 2015). Trichomes are mostly acidic, while trichome-bearing plant material is mostly carbohydrates. This difference in electric charge allows the separation of the trichome fraction in a strong electric field, using Coulomb forces. However, it should be noted that efficient separation requires decapitation and additional charging of the mixed powder.

**Fig. 2:**
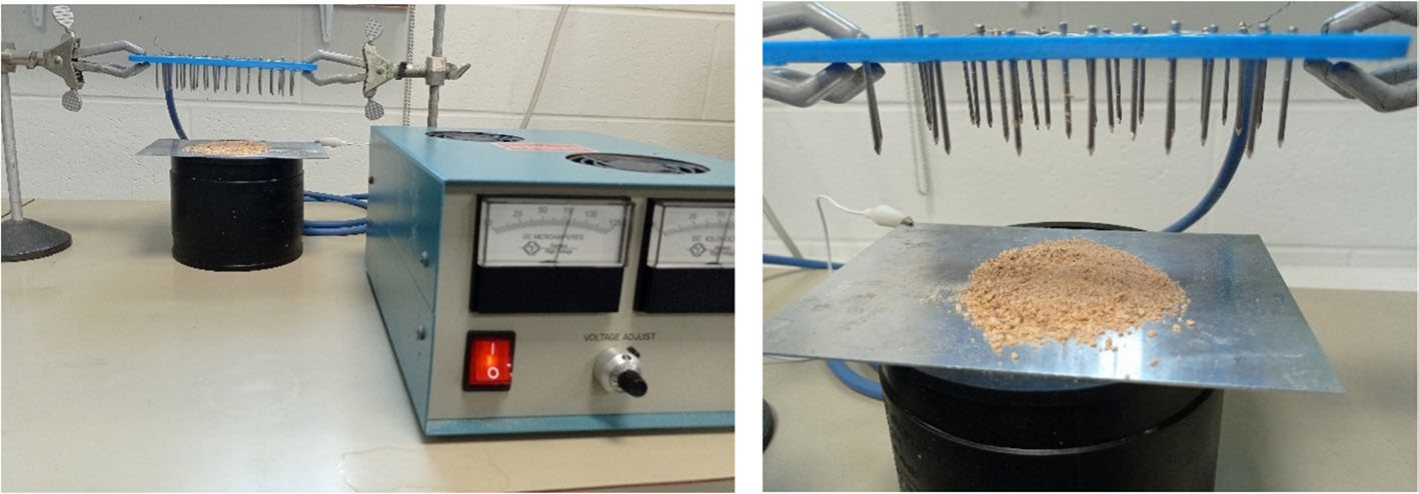
Corona charging of plant biomass

As an alternative to the manual operation of triboelectric charging on a nylon sieve, we explored the corona charging of grounded cannabis samples by the ionic wind (Fig. 2).

It was found that charging plant biomass by the ionic wind did not change the negative charge of trichomes but induced a positive charge on the stalk and chlorophyll-containing leafy fraction. This difference can facilitate the electrostatic separation of trichomes from trichome-bearing plant material. These electrical properties determine the different behaviour of particles in an electric field and separability in free-falling mode.

Key equations governing the motion of particles in an electrostatic field include Coulomb’s law and Newton’s second law, which determine the force exerted on charged particles and their subsequent acceleration, respectively. The equations provide a framework for optimizing separator designs for specific materials and applications. The next section analyses fundamental forces acting on particles in electrostatic and aerodynamic fields. The calculation of forces acting on typical cannabis powder allows evaluation of their effect on the particles.

### Fundamental forces

**Fig. 3:**
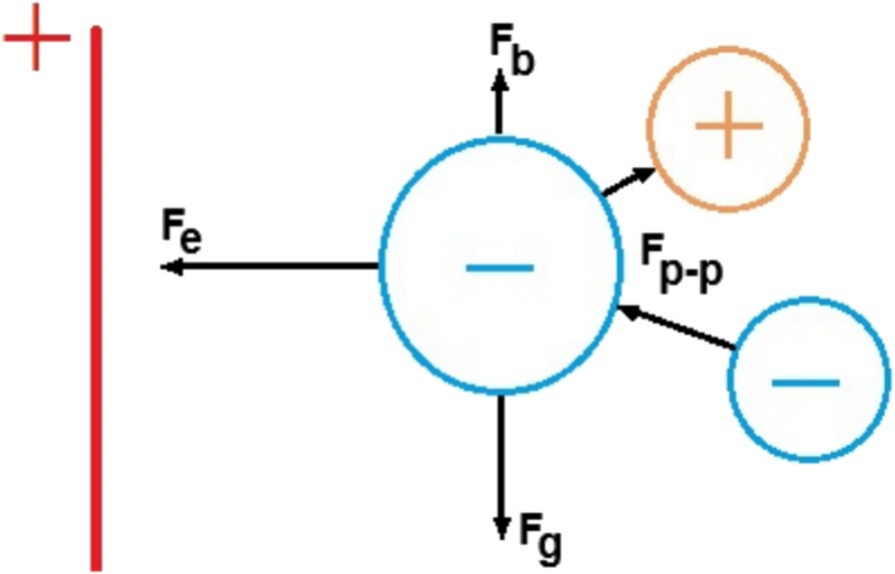
Forces acting on the charged particle in the electrostatic field

The review of fundamental forces acting on a free-falling particle in an electrostatic field is presented by Kelly and Spottiswood (1989b). The starting point of analysis is the electric properties of particles. Along with electrical conductivity and natural charge, polarizability or acquired charge plays an important role. Polarizability will determine the electric force acting on the particle F_e_, while density will determine the gravitational force F_g_. The size of the particle will determine the buoyancy force F_b_. We also need to consider particle–particle interaction F_p-p_, which depends on the particle charges. Negatively charged particles will attract positively charged particles and repel particles of the same polarity. A schematic picture of all acting forces is shown in Fig. 3.

Assuming the spherical shape of the charged particle, the electric force in the electrostatic field F_e_ is determined as:

2 $${F}_{e}=qE=4\pi {r}^{2}{\rho }_{e}E$$

where $r$ is the particle effective radius, m; ${\rho }_{e}$ is a specific charge, C/m^2^; and $E$ is electric field strength, V/m.

The gravitational force F_g_ depends on the material density ${\rho }_{m}$:

3 $${F}_{g}=mg=\frac{4}{3}\pi {r}^{3}{\rho }_{m}g$$

Buoyancy force F_b_ depends on the particle velocity $\upsilon :$

4 $${F}_{b}=\frac{1}{2}{\rho }_{a}{C}_{D}A{\upsilon }^{2}$$

where $A$ is a particle cross-sectional area, m^2^, ${\rho }_{a}$ is the air density, kg/m^3^, and ${C}_{D}$ is a drag coefficient.

The particle–particle force F_pp_ depends on the particle's charge ${q}_{1}{, q}_{2}$ and described by Coulomb's law:

5 $${F}_{pp}={k}_{e}\frac{{q}_{1}{q}_{2}}{{d}^{2}}$$

where $d$ is the distance between particles, m; and ${k}_{e}$ is an electrostatic constant (${k}_{e}$= 8.99 × 10^9^ N/m·C^2^).

This preliminary analysis of major electrostatic forces allowed the conceptual design of an electrostatic separator for cannabis trichomes extraction.

## Results

### Charge measurements

Charge measurements showed a linear correlation between sample charge and volumetric fraction of trichomes. Plant biomass had a positive charge, while samples with trichomes had a negative charge ranging from 20 to 100 pC. The charge was temperature-dependent, decreasing to 0 at temperatures below zero (−18 °C). Hence, electrostatic separation should be done at room temperature. It was also found that triboelectric charging of the sample by friction on the nylon sieve increased the negative charge of trichome heads and induced a positive charge on the stalk and chlorophyll-containing leafy fraction. The correlation between cannabinoid content and the charge was observed even before the decapitation of trichomes. Thus, electric charge could be a reliable indicator of the cannabinoid content in raw material. On the other hand, the trichome electric charge allows the calculation of forces acting on the particle in the electrostatic field.

### Determination of forces

The electrostatic force, created by the electrode system, was calculated from Eq. (2). With the typical size of a particle as 200 µm and a specific charge of 0.26 µC/m^2^ (Wang et al. 2014) the particle in the electrostatic field 3 × 10^5^ V/m experiences the force ${F}_{e}$~10^–6^ N. For smaller particles 70 µm, the electric force decreases tenfold to 10^–7^ N.

The gravitational force F_g_ was calculated using Eq. (3). There is almost a threefold difference in density between trichome heads and stalks ${\rho }_{heads}$=308 kg/m^3^ and ${\rho }_{stalk}$= 118 kg/m^3^, which determines the difference in gravitational force acting on the particles. The gravitational force for trichome heads is estimated as (0.12…0.005) × 10^–7^ N, while for stalks it is three times smaller—(0.04…0.002) × 10^–7^ N.

The buoyancy force F_b_ was estimated using Eq. (4). Considering the laminar flow velocity of 0.1 m/s and the drag coefficient ${C}_{D}$ equal to ~ 1.5, the calculated value of the buoyancy force is ~ 3 × 10^–10^ N, which is significantly smaller than the electric and gravitational forces.

The particle–particle force F_pp_ was evaluated from Eq. (5), for the average particle charge of 20 pC and the average distance between particles of 1 mm. The calculated value of particle–particle force is about 3.6 × 10^–6^ N. This value is comparable to the electrostatic force, indicating its significant role in clustering. This force could be smaller if the particles with opposite charges are uniformly distributed. In this case, the mechanical scattering of particles with a narrow diffuser could minimize the interaction effect.

In summary, the two dominant forces are the electric force from the external electric field F_e_ and the particle–particle force F_pp_. The separability of material ${\varvec{S}}$ depends on the ratio between the two forces:

6 $$S=\frac{{F}_{e}}{{F}_{pp}}$$

**Fig. 4:**
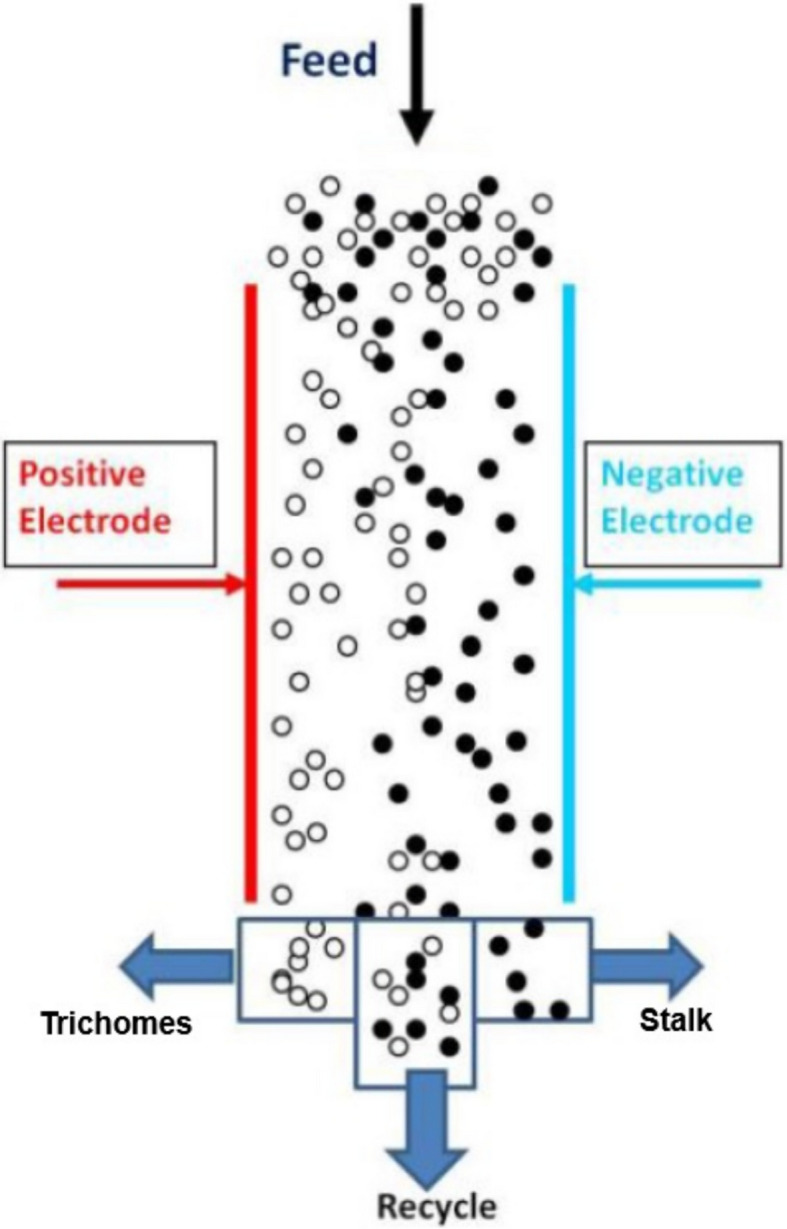
The principle of trichome separation in the electrostatic field

The electrostatic separator was designed to maximize ${\varvec{S}}$ by minimizing the effect of particle–particle interaction and maximizing the impact of the applied electric field. The process of hash separation is schematically shown in Fig. 4.

Particles are separated based on their trajectories, which depend on their mass, charge, and interaction with the electric field. Conductive particles quickly lose charge upon contact with grounded surfaces, while non-conductive particles retain their charge longer, enabling effective separation. Dry plant biomass is non-conductive. The particles with a negative charge (mostly trichomes) are attracted to the positive electrode, while particles with a positive charge (mostly stalk) are attracted to the negative electrode. After accumulation on electrodes, these fractions are scraped into separate bins. A part of the material did not acquire charge or stick in the conglomerate. This part goes through the separation chamber into the recycle bin and could be further fractionated by recirculation in the loop. The fraction of trichomes in the recycle bin indicates the efficiency of separation or separability.

The electric force is controlled by high voltage, applied between two electrodes. The flow rate of the material is controlled pneumatically by an airflow regulator and a vacuum device.

### Prototype design

**Fig. 5:**
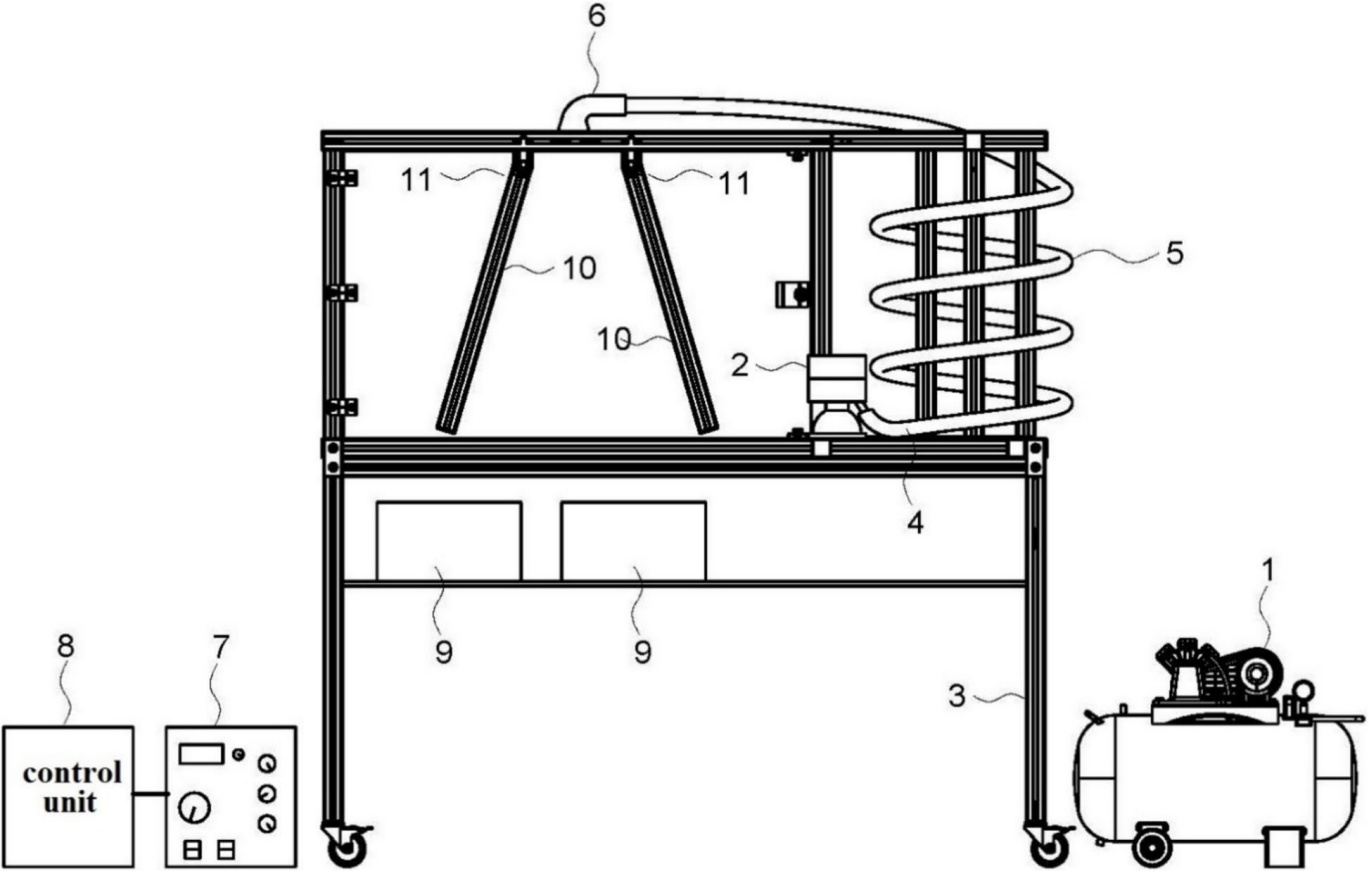
A sketch of a basic electrostatic separation unit. 1. Air compressor. 2. Vibrating feeder. 3. Separation chamber. 4. Airflow regulator. 5. Coiled pipeline. 6. Flow straightener (diffuser). 7. High-voltage power source. 8. Control unit. 9. Collection bins. 10. Electroconductive plate electrodes. 11. Insulators

A basic electrostatic separator unit consists of a feeder, charging system, separation chamber, collection system and control system. Figure 5 shows a separation chamber with a triboelectric charging system which consists of air compressor 1, vibrating feeder 2, airflow regulator 4, and coiled pipeline 5. The ground cannabis material with particle size in the range from 20 to 300 microns is manually or automatically supplied to vibrating feeder 2 and then pneumatically introduced by airflow to the coiled pipeline 5. The powder supply is controlled by airflow regulator 4, which may be manually controlled or actuated through an electronic signal.

Triboelectric charging of particles occurs in the coiled pipeline (5). Due to the different chemical composition, plant biomass particles acquire a positive charge, while trichomes acquire a negative charge. Immediately after triboelectric charging in the pipeline, charged particles are injected into the separation cabinet through a diffuser (6) with a narrow (about 3 mm) opening. Further separation of trichomes from the plant material is based on the difference in their electrostatic charge. Particles fall in the strong electric field between two electroconductive plate electrodes (10). The electrodes are oppositely charged from a high-voltage power supply 7, controlled through the control unit 8. The high-voltage source provides an electric field inversely proportional to the distance between electrodes. Both electrodes 10 are insulated using dielectric material 11 to prevent an electrical arc to the grounded metal frame of the separation chamber. Both electrodes are secured to the top of the frame. Non-parallel orientation of electrodes is necessary to create a higher gradient of electric field at the inlet, decreasing downstream the pathway of charged particles.

A higher electric field at the inlet applies additional force on the charged particles, resulting in a distortion of their trajectory from a vertical free-falling path. Downstream the electric field is decreasing, which results in the attraction of negatively charged particles to the positively charged electrode, while positively charged plant material is attracted to the negatively charged electrode. Uncharged or lost charge plant material falls into collection bins 9.

The innovation is based on the ability of an electrostatic separator to isolate the trichomes from plant biomass based on their unique electrochemical properties. It should be noted that electrostatic separation is not sensitive to the initial concentration of trichomes in plant material and could work with different grades of plant biomass. The spiral configuration of the feeding pipeline allows distributed and uniform exposure of the entire volume of the plant mixture to triboelectric charging. The turbulent regime of dry air prevents the clogging of charged particles. The ability of particles to acquire the charge can be regulated by the degree of throttling of a vibrating dispenser.

The diffusor with a narrow lateral nozzle provides better diffusion of particles in air volume between electrodes, which increases their exposure to the electric field during settling in the gravitational field. Long vertical electrodes provide sufficient exposure of particles to the electric field and therefore, a better separation effect. The trichomes are collected directly from the surface of the charged electrode. However, the manual collecting of trichomes limits the throughput capacity of the electrostatic separator. This deficiency could be eliminated by introducing self-cleanable electroconductive electrodes.

Self-cleanable electrodes contain a moving electroconductive belt, a rotating part, a mechanical drive (motor), and a transmission. The speed of the belt is determined by the accumulation rate of charged particles on the belt surface and controlled by the motor. This design of electrodes will allow full automation of the separation and collection of trichomes.

## Experimental validation

Cannabis plants collected from the field were dried to a moisture content of 0.06–0.15 g/g dry matter. Dried flowers were separated from stems, stalks, and branches using manual or mechanical methods. Dry plant biomass containing glandular trichomes was sifted using a rotary vibrating sieve or similar device (screened trommel) with an upper 250-micron stainless steel mesh and a lower 74-micron mesh screen. The result is a powder colloquially known as kief or hash. It contains glandular and other types of trichomes, pistils, trichome stalks, pollen, dirt, and fine particles of plant material with particle size distribution from 74 to 220 microns. Initial mechanical treatment on the sieve resulted in the detachment and liberation of most of the glandular trichome heads from the attached stalk, which is critical for further electrostatic separation, mostly determining yield and product purity.

Once the trichomes were liberated, they were processed in the electrostatic separator. The powder is fed into the device, controlling for optimal performance, air flow rate, position and angle of electrodes, temperature, humidity, and powder feed rate. Two fractions are created—a *heads* fraction containing mainly glandular trichome heads and a *tails* fraction containing mainly undesired contaminants. Powder that did not manage to separate is immediately reprocessed until all the powder is separated.

Fractions were checked under a microscope to evaluate purity and to determine the nature of the purity or contaminants. If feasible, heads and/or tails are returned to the vibrating screen for further liberation. Fractions were periodically examined under a microscope to determine if liberation was complete. This process can be repeated as necessary to achieve the desired purity. The final product is ready for post-processing or sold as a final product.

The comparison with conventional methods showed a first-pass average purity of 40–50%, and 50–60% if using a trommel screen type separator alone. In contrast, when the trommel is paired with the Plasmastatic separator, a first-pass purity of 80–95% round trichomes was achieved. A higher yield of about 98% trichomes is achievable in 2 or more passes.

## Discussion

Compared to conventional wet and dry fractionation, electrostatic separation of cannabis trichomes with Plasmastatic offers multiple advantages.

### High purity

Enables the selective isolation of trichomes with minimal contamination from plant material. Moreover, purity could be improved by fine-tuning electrostatic and aerodynamic fields. Another option is multiple recirculations of the material. In the first run, the trichome fraction could be partially (up to 20%) contaminated with the plant biomass. This material, with a purity of 80–95%, could be used for medicinal and cosmetic purposes. For special purposes, an electrostatic separator allows further purification to 99.8% due to multiple cycles or multiple separation stages.

### Non-destructive

Due to the minimal mechanical action, it preserves the integrity of trichomes. Short processing time preserves the quality of bioactive compounds. Oxidation of plant material could be further reduced by electrostatic separation in an oxygen-free environment (for example, helium or nitrogen).

### Solvent-free

Eliminates the need for chemical solvents, reducing environmental impact and production costs. Also, solvent-free technology minimized the damage to trichomes and the loss of water-soluble bioactive compounds.

### Scalability

Suitable for both small-scale and industrial applications. The separation rate of Plasmastatic is 0.5 kg/hour; but can be scaled to 100 kg/hour or more. It could be achieved due to increased surface of the electrode system, adjustment of particle flow rate, or a parallel/series combination of basic electrostatic units. The number of electrostatic separation units connected in parallel can achieve the desired throughput separation capacity. With the series configuration of basic electrostatic separation units, the higher purity of the product could be achieved. Due to extremely low energy consumption, electrostatic separators could be integrated with renewable energy sources, such as solar or wind energy in remote locations or mobile applications.

### Energy efficiency

It is a non-thermal technology, requiring much less energy compared to traditional methods like solvent extraction. The total energy used for the pilot-scale electrostatic separation is about 20 W for the separation throughput of about 100 g per minute. The energy efficiency of electrostatic separators is increasing with the increase of processing capacity tons per hour.

### Challenges and limitations

Despite high upfront investment for equipment and system setup, the electrostatic separator requires minimal maintenance costs. However, the operation requires trained operators to manage the process and troubleshoot issues. The initial preparation stage requires precise drying, grinding and liberation of trichomes from the cannabis material. Also, the electrostatic separator must meet regulatory compliance for safety and quality standards in the cannabis industry, which could differ in different countries. International patents protect the proprietary design of electrostatic separators. The good news is that the first models of electrostatic separators of cannabis trichomes have been on the market since 2023 under the brand “Plasmastatic” and are commercially available from SC Filtration (www.sambocreeck.com).

## Conclusions

Electrostatic separation technology marks a fundamental shift in cannabis postharvest processing. This technology may revolutionize the cannabis extraction industry by providing a cleaner and more efficient method for trichome isolation. By leveraging differences in electrical properties, this technique offers a high-purity, environmentally friendly, and scalable solution for cannabis extraction. So far, it is the only technology enabling continuous, streamlined extraction of trichomes from plant biomass. Therefore, this technology is suitable for the large industry's needs. Currently, the technology has reached TRL7 level, when the system prototype is successfully working in the operational environment. The next step in the deployment of electrostatic separation is certification and scaling to the desired industrial capacity. As the industry grows, adopting electrostatic separation will be key to meeting the demand for high-quality cannabis products.

Future research and development should focus on optimizing system design, improving throughput, and integrating this technology with downstream processing methods. Additionally, advancements in material science and electric technologies could further enhance separation efficiency and scalability.

## Data Availability

No datasets were generated or analysed during the current study.
